# Breath of Life: Heart Disease Link to Developmental Hypoxia

**DOI:** 10.1161/CIRCULATIONAHA.121.054689

**Published:** 2021-10-26

**Authors:** Dino A. Giussani

**Affiliations:** Department of Physiology, Development, and Neuroscience; The Barcroft Centre; Cambridge Cardiovascular British Heart Foundation Centre for Research Excellence; and Cambridge Strategic Research Initiative in Reproduction, University of Cambridge, UK.

**Keywords:** fetus, hypoxia, mitochondria, oxidative stress

## Abstract

Heart disease remains one of the greatest killers. In addition to genetics and traditional lifestyle risk factors, we now understand that adverse conditions during pregnancy can also increase susceptibility to cardiovascular disease in the offspring. Therefore, the mechanisms by which this occurs and possible preventative therapies are of significant contemporary interest to the cardiovascular community. A common suboptimal pregnancy condition is a sustained reduction in fetal oxygenation. Chronic fetal hypoxia results from any pregnancy with increased placental vascular resistance, such as in preeclampsia, placental infection, or maternal obesity. Chronic fetal hypoxia may also arise during pregnancy at high altitude or because of maternal respiratory disease. This article reviews the short- and long-term effects of hypoxia on the fetal cardiovascular system, and the importance of chronic fetal hypoxia in triggering a developmental origin of future heart disease in the adult progeny. The work summarizes evidence derived from human studies as well as from rodent, avian, and ovine models. There is a focus on the discovery of the molecular link between prenatal hypoxia, oxidative stress, and increased cardiovascular risk in adult offspring. Discussion of mitochondria-targeted antioxidant therapy offers potential targets for clinical intervention in human pregnancy complicated by chronic fetal hypoxia.

In the United Kingdom, 1 in 4 people die of cardiovascular disease, averaging 460 deaths each day or 1 every 3 minutes. This costs the United Kingdom more than £30 billion per year in patient care and lost workforce because of heart disease.^1^ Therefore, there is no question that cardiovascular disease is an important and expensive problem, imposing a significant burden on every nation’s health and wealth. We accept that our genetic make-up interacts with traditional lifestyle risk factors, such as smoking, obesity, or a sedentary life, in triggering a risk of cardiovascular disease. In addition, it has become established that the gene–environment interaction before birth may be as—if not more—important in setting a future risk of heart disease through a process known as developmental programming.^2^ This concept supports that the environment experienced around conception or during the fetal and neonatal periods permanently alters the anatomy and physiology of key organs and systems in offspring, thereby leading to an increased risk of cardiovascular disease later in life.^2^ Developmental programming is an extension of the Barker hypothesis, proposed in the late 1980s by British epidemiologist David Barker, who stated that intrauterine growth retardation, low birth weight, and premature birth, acting as surrogate markers of adverse intrauterine conditions, have a causal relationship with the origins of hypertension, coronary heart disease, and non–insulin-dependent diabetes, in middle age.^3^ The concept of developmental programming makes good sense because our physiology (ie, what makes us function) is much more malleable and plastic during early life. Therefore, periods during development relative to those in adulthood can be exquisitely sensitive to environmental conditions, yielding later-life traits shaped by early-life conditions through what has been described as developmental plasticity.^4^ One excellent example is that in some reptilian species, ambient temperature during a critical period of embryonic development can determine whether an egg develops as male or female.^5^ Thus, the magnitude of the effect of the environment on us across the life-course clearly varies, and it can be represented by a reverse exponential relationship having its maximal expression during embryonic or fetal life and diminishing progressively as we grow older (Figure 1). Equally important is that the opportunity for correction follows a similar trajectory: it is greatest in early life and diminishes progressively as we grow older (Figure 1). Therefore, in medicine and in public health today, there is a growing change in perception as to how to best tackle cardiovascular disease. Focus is moving away from treatment, where we can do comparatively little, toward prevention, where we can comparatively do a lot (Figure 1). It is clear that the earlier in development, the greater the window for intervention. Therefore, there is great interest in halting the progression of disease at its very onset; that is, if possible, to bring “preventative medicine back into the womb,” or to think of even earlier interventions that could take place during the preconceptual period.

**Figure 1.**
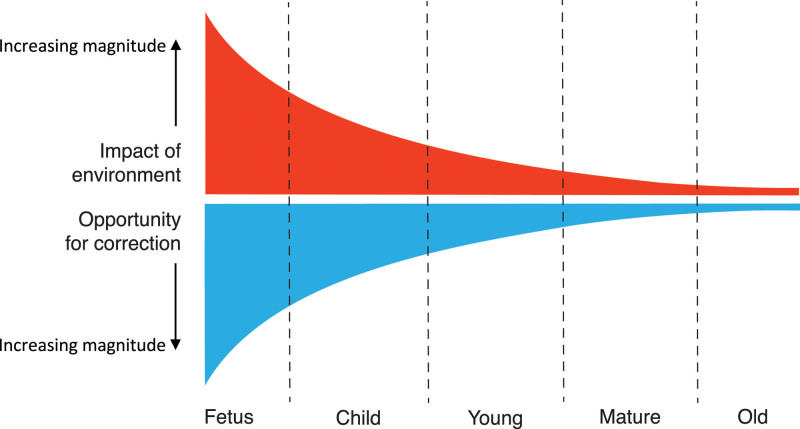
**Biological programming and age.** Laws of nature predict that the younger we are, the greater the impact the environment has on us. This environmental impact has its maximum expression during embryonic or fetal life, and it diminishes progressively as we grow older. Equally important is that the opportunity for correction follows a similar trajectory, being greatest in early life and diminishing progressively as we grow older. Therefore, in medicine and public health today, there is a growing change in practice away from treatment, when we can do comparatively little, toward prevention, when we can do comparatively a lot. Clearly, there is no better form of preventative medicine than bringing this concept right back along the developmental trajectory, when the window of opportunity for correction is greatest, and try to halt the development of disease at its very onset, by bringing “preventative medicine back into the womb.”

In humans, the best available evidence to support the concept of developmental programming comes from studies in women who were obese during a first pregnancy, lost weight through bariatric surgery, and were leaner during a second pregnancy.^6^ These studies show that siblings born before the bariatric surgery have signs of an increased cardiovascular risk (obesity, insulin resistance, and raised arterial blood pressure), compared with those born after surgery.^6^ Therefore, such studies in humans highlight that a different environment in the same womb can program a differential risk of cardiovascular disease in offspring of the same family. This provides compelling evidence in humans that the environment experienced during critical, sensitive periods of early development directly influences long-term cardiovascular health. Therefore, in this field of science, there is interest in how adverse conditions during pregnancy may program an increased cardiovascular risk in the adult offspring and, if so, through which mechanisms, so that we can design interventional strategies.

In recent years, many excellent reviews have discussed the effect of pregnancy complicated by changes in maternal nutrition, exposure to stress hormones, environmental pollution, or maternal substance abuse on programmed cardiovascular disease in the offspring at adulthood.^7–10^ By contrast, the contribution of pregnancy complicated by chronic hypoxia has been comparatively ignored. This is surprising, given that chronic fetal hypoxia is one of the most common outcomes of complicated pregnancy in humans.^11^ In essence, any condition that increases placental vascular resistance will decrease oxygen delivery to the growing baby (eg, during preeclampsia, placental insufficiency, or chorioamnionitis).^11,12^ It is important to note that inflammatory conditions during pregnancy are quite topical today; gestational diabetes and maternal obesity are just as concerning, since they also promote an increase in placental vascular resistance, thereby leading to chronic reductions in fetal oxygenation.^11–13^ Therefore, chronic fetal hypoxia provides a common path for several complications in pregnancy, and there is accumulating evidence derived from human studies as well as animal models to support that it can trigger an early origin of cardiovascular dysfunction and program susceptibility to cardiovascular disease in later life. To understand how fetal hypoxia may program an increased cardiovascular risk, we must first consider the short- and long-term effects of hypoxia on the fetal cardiovascular system.

## Hypoxia: Short-Term Effects on the Fetus

### From Starling to Bohr

**Figure 2.**
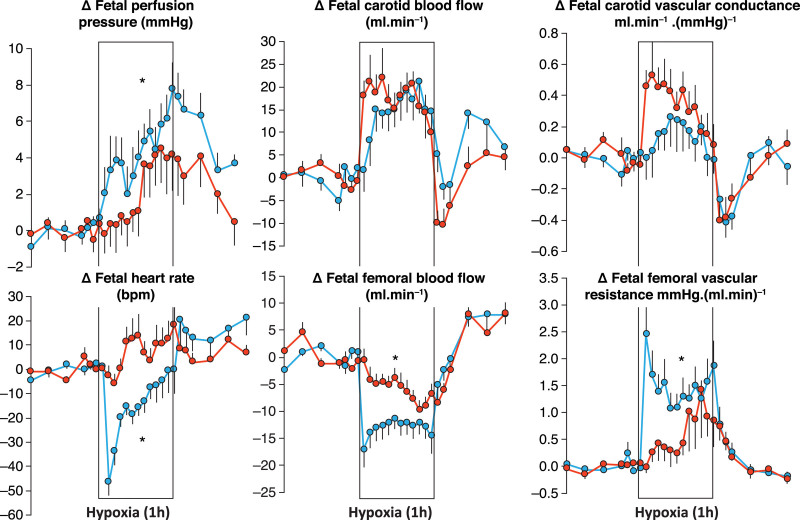
**Cardiovascular defense to acute hypoxia.** Values are mean±SEM for the change from baseline in perfusion pressure (arteriovenous difference), heart rate, carotid and femoral arterial blood flow, carotid vascular conductance (flow/pressure) and femoral vascular resistance (pressure/flow) during a 1-h period of acute hypoxia (box) in 14 intact (blue circles) and 12 carotid chemoreceptor denervated (red circles) fetal sheep in late gestation. The Darcy law of flow, which is the hydraulic equivalent of the Ohm law of electricity, was formulated in 1856 after his study of water flowing through gravel beds of the fountains of Dijon: flow in the steady state is linearly proportional to the pressure difference between 2 points and inversely proportional to the resistance across them. Vascular conductance and resistance can be calculated by applying the Darcy law to the circulation: blood flow is proportional to the arteriovenous pressure difference and inversely proportional to vascular resistance. Carotid chemoreceptor denervation attenuates the increase in fetal perfusion pressure, abolishes the fetal bradycardia, and delays the fall in femoral blood flow and the increase in femoral vascular resistance without affecting the increase in carotid blood flow or vascular conductance in response to acute hypoxia. *P<0.05, intact vs carotid chemoreceptor denervated by ANOVA. Redrawn from Giussani et al^16^ with permission. Copyright ©1993 The Physiological Society.

In contrast with life after birth, during which periods of hypoxia can be compensated for by an increase in oxygenation secondary to an increase in lung ventilation, the fetus adopts a different strategy to withstand episodes of reduced oxygenation. As the fetus obtains oxygen from the mother through the placenta; upon reduction in oxygenation, the fetus can decrease oxygen consumption, increase oxygen extraction, and/or make best use of the available oxygen supply.^14–16^ The fetal cardiovascular defense responses to hypoxia exemplify all of these strategies (Figure 2). In response to acute hypoxia, there is a reduction in fetal heart rate, which is useful for several reasons. Fetal bradycardia reduces myocardial oxygen consumption, and prolongs the beat-to-beat interval, thereby lengthening end-diastolic filling time and, hence, increasing end-diastolic volume.^11,14^ The latter increases the stretch on the fetal myocardial cells, increasing their tension through greater interaction between the thick and thin myofilaments. As a result, the force of contraction in the fetal myocardium is increased via the Frank–Starling mechanism, which helps maintain cardiac output, despite bradycardia.^11,14^ The fall in heart rate and prolongation of the beat-to-beat interval also reduces the velocity of blood flow through the microvasculature, allowing longer time for oxygen extraction by tissues that are becoming hypoxic.^11,14^ In clinical practice, the fetal heart rate response to acute hypoxia is indicated by fetal heart rate decelerations and it is a response exploited every day in fetal heart rate monitoring when assessing fetal well-being during the uterine contractions of labor, which can restrict uteroplacental blood flow and reduce fetal oxygenation.^15^

Hypoxia also affects the fetal circulation. During acute fetal hypoxia, there is a redistribution of the fetal cardiac output, shunting blood flow away from the peripheral circulations toward the fetal brain, heart and adrenal glands.^11,14^ This circulatory defense has been dubbed as “the fetal brain sparing response” and in animal models, such as in late-gestation fetal sheep, it is reflected by an increase in carotid blood flow and a fall in femoral arterial blood flow (Figure 2).^16^ Applying Ohm's law to fluid dynamics, the French engineer Darcy stated that, in the steady state, flow is linearly proportional to the pressure difference between 2 points and inversely proportional to resistance across them.^17^ Applying Darcy's Law to the circulation, this suggests that blood flow is proportional to the arteriovenous pressure difference and inversely proportional to vascular resistance. Therefore, whether the increase in carotid blood flow in the fetus during acute hypoxia is purely pressure-driven—or greater than can be accounted for by an increase in fetal arterial blood pressure—can be approximated by calculating carotid vascular resistance (fetal arterial pressure/fetal carotid blood flow), or better still carotid vascular conductance, which is the inverse of resistance (fetal carotid blood flow/fetal arterial blood pressure). This calculation reveals a significant increase in carotid vascular conductance in the fetus during acute hypoxia (Figure 2),^16^ meaning that improved cerebral blood flow in the fetus during acute hypoxia is not a passive process purely driven by an increase in fetal arterial blood pressure. Rather, there is active vasodilatation in the fetal cerebral microvasculature during acute hypoxia, which is known to result from local increases in many dilator agents, including adenosine, nitric oxide, and prostanoids.^18^ Similarly, the fall in fetal femoral arterial blood flow during acute hypoxia is secondary to active peripheral vasoconstriction. Jean Léonard Marie Poiseuille stated that halving the radius of a tube will increase its resistance by 16 times.^19^ Hence, the femoral vasoconstriction in the fetus during acute hypoxia will decrease the lumenal radius of the femoral artery, triggering a marked increase in femoral vascular resistance (fetal arterial blood pressure/fetal femoral arterial blood flow).^16^ Consequently, during an acute episode of oxygen deprivation, the fetal cardiac output follows the path of least resistance, and blood flow is prioritized away from less essential vascular beds to maintain oxygen delivery to more hypoxia-sensitive regions, such as the developing brain, heart, and adrenal glands.^11^ In obstetric practice, this hemodynamic redistribution in the fetus is indicated by changes in Doppler blood flow velocimetry, whereby an increase in regional vascular resistance is picked up by an increase in the pulsatility index of the waveform of the vascular bed in question. Hence, clinically, a fall in the Doppler pulsatility index of the fetal middle cerebral artery relative to the umbilical artery flow velocity waveform is a sign of fetal hypoxia and fetal brain sparing.^20^

Oxygen delivery is coupled to oxygen consumption. Therefore, limiting blood flow to less essential vascular beds, such as the fetal gut and fetal hind limbs, during the fetal brain-sparing response also contributes to an overall fall in fetal oxygen consumption during acute hypoxia.^21^ Decreased oxygen delivery to the fetal hind-limb musculature leads to anaerobic metabolism, increasing lactate output and acidifying the fetal blood.^21^ This not only facilitates the unloading of oxygen from hemoglobin to the fetal tissues through a Bohr effect; the fall in fetal pH also sensitizes the fetal cardiovascular defense to acute hypoxia, enhancing the magnitude of the fetal brain-sparing circulatory responses.^22,23^

### Mechanisms Underlying the Fetal Cardiovascular Defense to Acute Hypoxia

**Figure 3.**
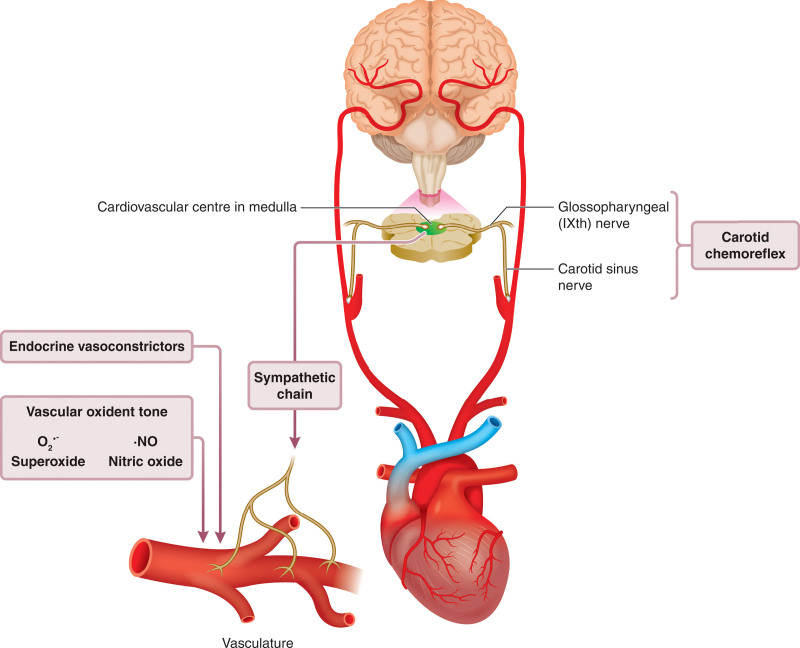
**Physiology underlying the fetal cardiovascular defense to acute hypoxia.** Activation of a carotid chemoreflex by hypoxic blood triggers a fall in heart rate and vasoconstriction in the peripheral circulation in the fetus. The fall in heart rate is vagally mediated and the peripheral vasoconstriction is initiated by activation of the sympathetic nerves. Vasoconstrictor hormones, such as catecholamines and vasopressin are then released into the fetal circulation to maintain the neurally-triggered vasoconstriction. The neuroendocrine vasoconstriction is then supplemented by a vascular oxidant tone, which is promoted by an increase in the ratio of free radicals, such as the superoxide anion (O2^•-^) relative to nitric oxide (NO), both of which are increased in the fetus during periods of oxygen deprivation. The magnitude of the peripheral vasoconstrictor response to acute hypoxia in the late-gestation fetus therefore depends on the partial contributions of chemoreflex, endocrine, and local vascular redox responses. Redrawn from Giussani^11^ with permission. Copyright ©2016, The Physiological Society.

The physiology underlying the fetal cardiovascular defense to short episodes of hypoxia is well delineated.^11^ We know that the cardiovascular responses are triggered exclusively by a carotid chemoreflex.^16^ Bilateral section of the carotid sinus nerves abolishes the fetal bradycardia and delays the increase in femoral vascular resistance without affecting the increase in carotid vascular conductance in response to acute hypoxia (Figure 2).^16^ After the increase in carotid chemoreceptor discharge to the fetal brain stem, there is activation of the autonomic nervous system increasing vagal outflow to the heart coupled with sympathetic outflow withdrawal, which reduces heart rate (Figure 3).^16^ There is also increased sympathetic outflow to the peripheral circulation, which promotes vasoconstriction via release of noradrenaline and neuropeptide Y acting on α-adrenergic and Y1 receptors, respectively.^16,24,25^ Once triggered, the peripheral constriction is maintained by the release of hormones into the fetal circulation, such as an increase in catecholamines and vasopressin.^26,27^ Chemoreflex and endocrine responses to acute hypoxia in the fetus mature with advancing gestational age toward term, in parallel with the prepartum surge in fetal plasma cortisol.^27,28^ Therefore, antenatal glucocorticoid therapy in pregnancy threatened by preterm birth can be exploited to accelerate the maturation of the fetal cardiovascular defense to acute hypoxia, as well as the fetal lungs.^28–32^

In the past few years, research has revealed that the neuroendocrine vasoconstriction triggered by the carotid chemoreflex and maintained by constrictor hormones in the fetus is supplemented by a third component, which is the vascular oxidant tone. This redox influence acts locally at the level of the microcirculation and is promoted by an increase in the ratio of free radicals, such as the superoxide anion (O_2_^•-^) relative to nitric oxide (NO), both of which are increased in the fetus during periods of oxygen deprivation.^33,34^ This vascular redox tone is operational in the late gestation fetus and it can be manipulated in favor of constriction or dilatation by altering the numerator or denominator of the O_2_^•-^:NO fraction.^34–37^ The magnitude of the peripheral vasoconstrictor response to acute hypoxia, an important contributor to the fetal brain-sparing response in the late gestation fetus, therefore depends on the aggregate contributions of chemoreflex, endocrine, and local vascular redox responses (Figure 3).^11^

## Hypoxia: Long-Term Effects on The Fetus

### Maintained Redistribution of Blood Flow

It has now become clear that the fetal hemodynamic response to oxygen deprivation, redistributing cardiac output toward the fetal brain, heart, and adrenals, can persist during pregnancy complicated by chronic fetal hypoxia.^11,12,38–40^ Although protective, this compensatory circulatory response is insufficient to prevent adverse outcomes in infants born from hypoxic pregnancies. For example, ≈50% of cases can show signs of adverse neurological outcomes, ranging from overt brain lesions, such as leukomalacia and cerebral palsy, to a wide range of more subtle disturbances, such as changes to the morphology of the retinal optic nerve.^41^ Even in milder cases, the maintained fetal redistribution of blood flow in response to chronic hypoxia may also trigger unwanted side-effects on fetal growth and cardiovascular development. Chronic hypoxia directly, and indirectly via elevations in fetal plasma catecholamine levels, will inhibit fetal insulin secretion.^42^ The increase in blood glucose availability will help its redistribution to the fetal brain, heart, and adrenal glands during chronic hypoxia, promoting a benefit.^40^ However, the trade-off will be a reduction in glucose uptake by other fetal tissues, which will decelerate fetal growth rate. Likewise, IGF-1 (insulin-like growth factor 1) is suppressed in virtually all models of fetal growth restriction.^7^ Further, fetal glycemia will reduce the maternal-to-fetal glucose concentration gradient, worsening substrate transfer for fetal growth.^43^ Recent evidence suggests that the growth-restricted human fetus in pregnancy is complicated by chronic hypoxia triggers compensatory mechanisms to reduce its metabolic rate, matching the proportion of substrate consumption relative to oxygen delivery as a survival strategy.^44^ Despite beneficial adaptations by the placenta, designed to lessen the negative effect of chronic hypoxia on fetal growth,^45^ these metabolic adaptations in the chronically hypoxic fetus coupled with the sustained redistribution of blood flow from the periphery results in asymmetrical fetal growth restriction. This yields offspring whom are thin for their length and/or have an appropriate head size relative to their comparatively shorter body length. In neonatology, this is indicated by a fall in birth weight with a reduced ponderal index or with an increase in the bi-parietal diameter relative to the body length ratio or with an increase in the head circumference relative to abdominal girth ratio of the new-born baby, all of which are clinical signs that the pregnancy has been complicated by chronic fetal hypoxia.^11^

**Figure 4.**
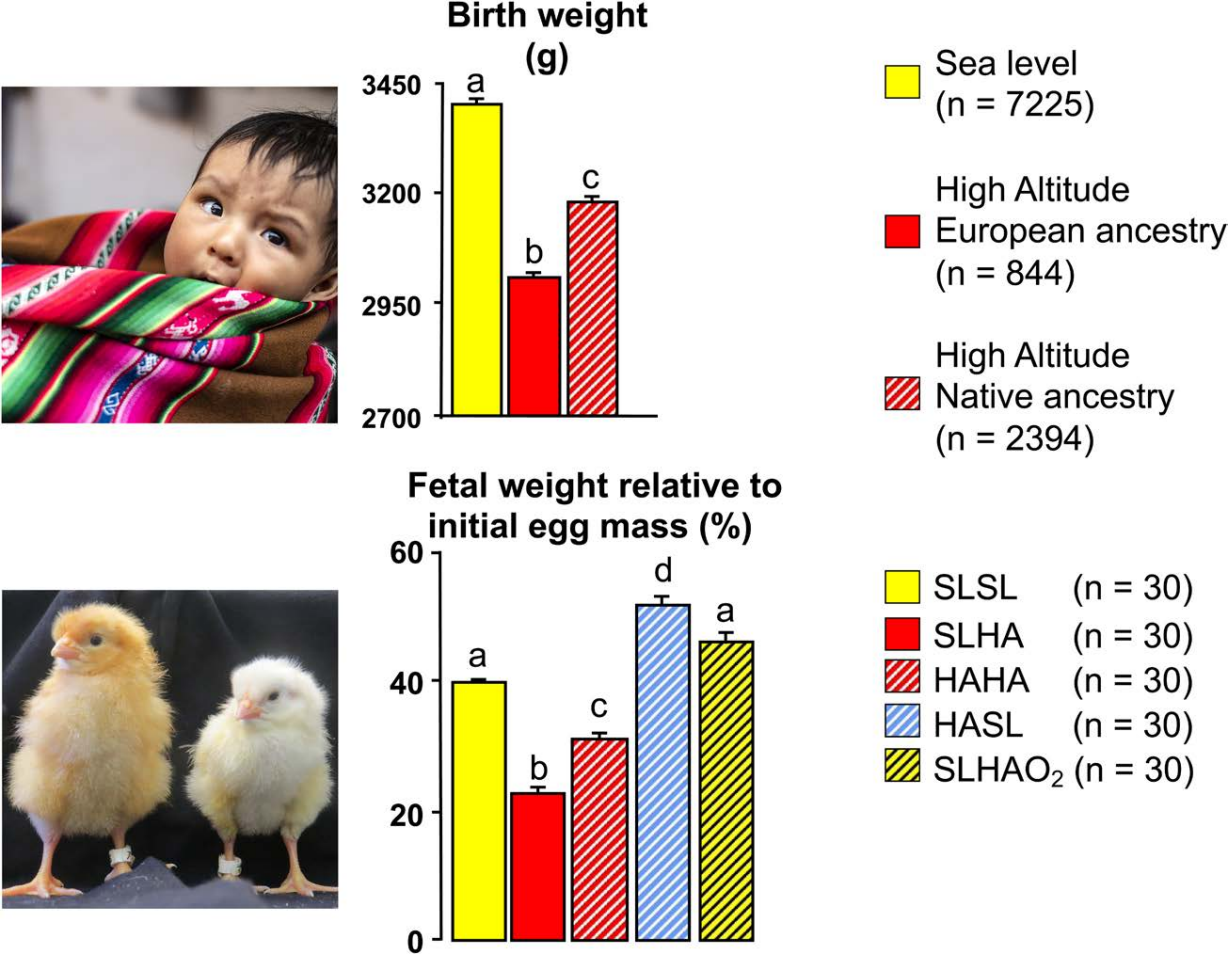
**The role of oxygen in the regulation of embryonic and fetal growth.** Studies in Bolivia have revealed that babies born in the high-altitude city of La Paz (**top**, red bars; ≈3600 meters above sea level) have lower birth weight compared with babies born at the sea-level city of Santa Cruz (**top**, yellow bar). However, babies born from Andean mothers in La Paz (**top**, red hatched bar) show protection against this effect, being significantly heavier than babies born from European mothers in La Paz (**top**, red solid bar). These discoveries have been replicated and extended by studies in the chicken embryo. Incubation in La Paz of fertilized eggs laid by sea-level hens (**bottom**, red solid bar) led to a significant reduction in embryo weight by the end of the incubation period when compared with incubation in Santa Cruz of fertilized eggs laid by sea-level hens (**bottom**, yellow solid bar). Incubation at high altitude in La Paz of fertilized eggs laid by highland hens which had lived in La Paz for generational times (**bottom**, red hatched bar) produced embryos that were heavier compared with incubation in La Paz of fertilized eggs laid by sea-level hens (**bottom**, red solid bar). This finding confirms the protection against the effects of high-altitude hypoxia on fetal growth by prolonged highland-residence ancestry discovered in humans. Incubation of fertilized eggs laid by high-altitude hens at sea level in Santa Cruz (**bottom**, blue hatched bar) prevented the expected reduction in embryo weight. In fact, this group of embryos not only recovered their growth potential, but grew heavier when compared with embryos from eggs laid in Santa Cruz by sea-level hens (**bottom**, yellow solid bar). This is despite high-altitude eggs being substantially smaller than sea-level eggs. Last, incubation of fertilized eggs laid by sea-level hens at high altitude with oxygen supplementation at rates to equate to sea-level Pao_2_ (**bottom**, yellow hatched bar) prevented the high altitude–induced reduction in embryo weight, confirming an isolated effect mediated by hypoxia. Values are mean±SEM. *n* numbers shown in brackets. Significant letters are significantly (*P*<0.05) different (ANOVA plus Tukey test). HAHA indicates high-altitude hens at high altitude; HASL, high-altitude hens at sea level; SLHA, seal-level hens at high altitude; SLHAO_2_, sea-level hens at high altitude with oxygen supplementation at rates to equate sea-level Pao_2_; and SLSL, sea-level hens at sea level. Data from Giussani et al^46,85^ with permission (Copyright ©2001, Springer Nature; Copyright ©2007, The Physiological Society) and Soria et al^47^ (Copyright ©2013, Springer Nature). Photos reprinted from Itani et al^97^ with permission.

Evidence in humans suggesting that oxygen tension is an important dictator of fetal growth is provided by the well-known effects of the hypobaric hypoxia of pregnancy at high altitude, which yields babies of low birth weight compared with those born at sea level, with clinical signs of asymmetry (Figure 4).^46,47^ Studies of obstetric records in Bolivia from healthy term human pregnancies showed that babies born in the high-altitude city of La Paz (≈3600 meters above sea level) were significantly lighter compared with those born in the Bolivian sea-level city of Santa Cruz, independent of effects imposed by socioeconomic status.^46,47^ It is interesting that babies born in La Paz from mothers of Andean ancestry, compared with those of European ancestry, were less growth restricted, despite the former showing greater indices of poverty, including maternal malnutrition.^46,47^ These discoveries reveal a protective trait against the effects of high-altitude hypoxia on fetal growth has developed through generations of residence at high altitude and it is strong enough to overwhelm any potential detrimental effects of even maternal undernutrition on fetal growth.^48^

Compared with asymmetrical fetal growth restriction, the lesser well-described adverse consequence of sustained fetal peripheral vasoconstriction is that it may lead to maintained increases in fetal cardiac afterload, ie, the force against which the fetal heart pumps. Persistent increases in fetal arterial blood pressure can remodel the cardiovascular system during sensitive periods of development and cause damage to blood vessels, leading to endothelial dysfunction and increased clotting, both of which are associated with an early origin of atherosclerosis.^49^ Endothelial dysfunction and cardiac and aortic wall hypertrophy are the harbinger of cardiovascular disease, ideally placed to increase the risk of future cardiovascular susceptibility to dysfunction, even before birth.^49^

### Programmed Heart Disease and Interventions

Given the significant effects of chronic fetal hypoxia in altering the physiology and anatomy of the developing cardiovascular system, attention has shifted to determine long-term consequences across the life course. Research has focused on the effects of hypoxic pregnancy on the cardiovascular health of the offspring at adulthood, aiming to isolate underlying mechanisms and thereby identify plausible interventions. The following sections summarized some of this research effort, focusing on human studies, and investigations in rodent, avian, and ovine species.

#### Insight From Human Studies

Evidence from human pregnancy is consistent with chronic fetal hypoxia affecting progeny through the life course. In humans, chronic fetal hypoxia triggers not only fetal growth restriction, but also a fetal origin of cardiovascular dysfunction, and programs an increased risk of heart disease in adult offspring. In the late-gestation human fetus, persistent increases in fetal cardiac afterload can remodel the fetal cardiovascular system, promoting thickening of the walls of the heart and major blood vessels.^50,51^ Other reported abnormalities in the growth-restricted human fetus include a decrease in cardiomyocyte volume^52^ and impaired cardiac ejection force and diastolic filling.^53^ By early childhood, offspring born growth-restricted show atheromatous alterations in the abdominal aorta,^54^ with persistent thickening of the aortic wall even when adjusted for their smaller body size^55^ and increased risk of systemic hypertension.^55^ Other studies have revealed a significant inverse relationship between low birth weight and endothelial dysfunction in 9- to 11-year-old children.^56^ Longer follow-up studies of men and women who were born growth-restricted similarly show that low birth weight is associated with endothelial dysfunction and systemic hypertension by young adulthood.^57,58^

In the setting of human pregnancy affected by fetal growth restriction, there have been some translational interventional studies, aiming to ameliorate deleterious effects on the mother and the offspring. Interventional clinical studies conducted during pregnancy have focused on the effects of maternal oxygen therapy and maternal exercise. Some studies conducted after birth have investigated early-life intervention strategies in children from fetal growth–restricted pregnancies, aiming to curtail possible adverse long-term cardiometabolic outcomes.

Studies of maternal oxygen therapy in pregnancy with suspected chronic fetal hypoxia have been reported for more than 30 years, but clear evidence of any benefit for mother or child remains to be determined. Reviews on this topic, including a Cochrane analysis, report on clinical studies that primarily focus on fetal blood gases and fetal growth in complicated (human) pregnancy.^59,60^ Despite some evidence supporting that maternal oxygen therapy can improve fetal oxygen levels and fetal aortic blood flow in severe early onset fetal growth restriction,^61^ to date, all studies have concluded that the value of maternal hyperoxygenation for the treatment of hypoxic fetal growth restriction remains to be established.^59–61^ In fact, some studies have highlighted potential adverse effects of maternal oxygen therapy by increasing levels of oxygen free radicals in the maternal and fetal circulation.^62^ Higher levels of oxygen free radicals will sequester nitric oxide and offset any potential benefit of maternal hyperoxygenation on fetal oxygen levels by increasing vascular resistance.^33–37^ Accordingly, it has been reported that maternal oxygen therapy can impair the increase in maternal cardiac output and diminish the fall in maternal peripheral vascular resistance, which are part of the normal maternal adaptive responses to pregnancy.^62^ Certainly, there have been no human clinical studies testing maternal oxygen therapy intervention in hypoxic pregnancy to protect against an increased risk of cardiovascular disease in the adult offspring.

Similarly, there has been interest in the potential beneficial effects of maternal exercise during pregnancy on the physiology of the mother and offspring for close to 2 decades.^63^ Prenatal exercise lowers pregnancy complications, including gestational diabetes, gestational hypertension, and maternal depression.^64^ A systematic review and meta-analysis on the subject concluded that prenatal exercise is safe and beneficial for the fetus, as it reduced the odds of macrosomia and was not associated with adverse neonatal or childhood outcomes.^65^ A second systematic review and meta-analysis on the effects of vigorous exercise in the third trimester of pregnancy reported that pregnant women who engaged in vigorous physical activity had a small but significant increase in the length of gestation and a small but significant decrease in the risk of giving birth preterm.^66^ However, whether maternal prenatal exercise protects fetal growth in hypoxic pregnancy or has any benefit against cardiovascular disease in adulthood programmed developmentally by prenatal hypoxia has again not been investigated.

Recognized postnatal interventions in early life that may protect the cardiovascular health of the child born from a pregnancy complicated by fetal growth restriction include the promotion of breastfeeding and intervention with diets rich in healthy fats.^51,67,68^ Children born from fetal growth–restricted pregnancies who are breastfed for at least 6 months have lower weight gain during childhood and improved cardiovascular remodeling.^51,67^ Consumption of diets rich in omega-3 fatty acids also conferred cardiovascular benefits, such as lower arterial blood pressure and protection against aortic wall thickening, in children who were born small for gestational age, but not to those born with normal birth weight.^51,68^

#### Insight From Rodent Models

Investigations in rodent species, including work using rats, mice, and guinea pigs, also support a link between pregnancy complicated by chronic hypoxia and an increased risk of cardiovascular dysfunction in the offspring. Several groups have reported that hypoxic pregnancy in rats programs endothelial dysfunction, hypertension, cardiac wall remodeling, and cardiac sympathetic dominance in adult offspring.^12,69–71^ There is strong evidence to support that the peripheral endothelial dysfunction in the adult offspring of hypoxic pregnancy is attributable to impaired nitric oxide bioavailability and signaling.^12,69–71^ It has been suggested that the programmed cardiac phenotype of hypertrophy and cardiac sympathetic dominance may represent an attempt to maintain cardiac output in the face of increased afterload.^70,71^ However, there is evidence that cardiac hypertrophy may be triggered independently of changes in fetal arterial blood pressure in some rodent models of adverse intrauterine conditions, as is the case in maternal obesity during pregnancy.^72^ Cardiac function that is sympathetically dominant, although adaptive, cannot be sustained in the longer-term and this cardiac phenotype has been linked with eventual heart failure and cardiovascular collapse.^70,71^ Consistent with this idea, investigations in rodents have also reported that hypoxic pregnancy increases susceptibility of the heart to ischemia–reperfusion injury at adulthood.^73^

Although several molecular pathways could link developmental hypoxia with programmed cardiovascular dysfunction in the adult offspring, including excess glucocorticoid exposure^8,31,32^ and activation of inflammatory cascades,^74,75^ the weight of the evidence derived from many independent studies in rodents supports oxidative stress having a major role.^70,71,76–78^ Chronic hypoxia increases markers of oxidative stress in the placenta and fetal organs, including the heart and vasculature,^12,70,71,76–78^ and maternal treatment in hypoxic pregnancy with antioxidants, such as vitamin C,^70,71,79^ allopurinol,^80^ or resveratrol,^12^ all protect against programmed cardiovascular dysfunction in adult offspring. While such interventional studies provide a robust proof-of-principle supporting that maternal treatment with antioxidants in pregnancy complicated by chronic fetal hypoxia can be protective, translation of these discoveries to the human clinical situation is riddled with complications and questions that require further clarification. First, several of these experimental studies, including ours, have induced a reduction in oxygenation by placing the pregnant rodent dam in a hypoxic chamber.^12,70,71,76–80^ This experimental approach is one of the few that permits exposure to chronic hypoxia of the embryo or fetus without physical obstruction of the uterine and/or umbilical circulation. While such an experimental design is important to best isolate the effects of developmental hypoxia in programming without significant perturbation in other nutrient transfer across the placenta, exposure of the pregnant dam to hypoxia leads to oxygen deficits in the maternal, placental as well as the fetal compartments. Therefore, the differential effects of hypoxia on the mother, placenta, or fetus and their partial contribution to programming cardiovascular dysfunction in the adult offspring remains uncertain. Second, in contrast with humans, mice and rats are born highly immature, where many maturational processes carry on into the postnatal period.^81^ Therefore, antioxidant treatment in these species during pregnancy may not equate to a similar timeline in human development. Third, rats and mice are polytocous species, giving birth to litters where the maternal metabolic adaptations to pregnancy are very different. Fourth, although studies in rodent hypoxic pregnancy with maternal antioxidant treatment with vitamin C proved to be protective, only very high doses incompatible with human clinical translation were effective.^70,71,79^ This is problematic, as randomized placebo-controlled human clinical trials have reported that concomitant supplementation with vitamins C and E does not prevent preeclampsia in women at risk but does increase the rate of babies born with a low birth weight.^82^ Therefore, in this field of science, there is an important need to isolate the partial effects of hypoxic pregnancy on the maternal, placental, and fetal compartments, as well as to design interventional studies using candidate antioxidants of improved clinical translation in animal models of comparable cardiovascular developmental milestones to humans.

#### Insight From Bird Studies

Avian species offer several interesting characteristics as an animal model for developmental programming studies.^83^ Since the embryo develops in isolation, studies in birds are ideal for disentangling the direct effects of adverse conditions on embryonic or fetal cardiovascular development from additional influences on the maternal or placental physiology. Additional advantages in chickens include their short incubation period, meaning that studies can be conducted more quickly; all available nutrition is fixed within the egg from the start of incubation; and there is no need for consideration of within litter variation or cross-fostering studies, as there are no effects of developmental conditions on lactation. Therefore, the model reduces the number of animals needed, abiding strongly to the 3R principle (replace, reduce, refine), enshrined by the Home Office EU Directive. In addition, the timing for many cardiovascular developmental milestones aligns more closely between chickens and humans compared with rodents.^84^ Further added value of research in the chicken embryo model is that candidate therapeutic agents can be administered topically via a small hole in the eggshell air cell onto the richly vascularized chorioallantoic membrane. Therefore, the chicken embryo model not only helps to isolate the direct effects of adverse developmental conditions, but also those of potential treatments, on the cardiovascular structure and function of the bird either during the incubation period or at adulthood.^83,84^

Studies using chicken embryos have revealed that the hypobaric hypoxia of life at high altitude can also reduce fetal growth and that this effect can be mitigated by prolonged residence ancestry at high altitude, as in humans (Figure 4). Incubation at La Paz of fertilized eggs laid by sea-level hens led to a significant reduction in embryo weight by the end of the incubation period.^85^ Conversely, incubation of fertilized eggs laid by sea-level hens at high altitude with oxygen supplementation prevented the high altitude–induced reduction in embryo weight. Further, incubation at high altitude in La Paz of fertilized eggs laid by highland hens which had lived in La Paz for generations produced embryos that were larger compared with those of fertilized eggs laid by sea-level hens and incubated in La Paz. This finding in chickens confirms the protection against the effects of high-altitude hypoxia on fetal growth by prolonged highland residence ancestry first discovered in humans.^46–48^ Last, incubation of fertilized eggs laid by high-altitude hens at sea level in Santa Cruz prevented the expected reduction in embryo weight. In fact, this group of embryos not only recovered their growth potential, but they grew comparatively heavier than embryos from eggs laid by hens at sea level, despite high-altitude eggs being substantially smaller than sea-level eggs (Figure 4).^85^ Combined, these studies in birds and humans highlight the important direct role of oxygen in regulating fetal growth when considered in isolation. Furthermore, the studies emphasize that highland-adapted individuals can confer protection against the effects of hypoxia on fetal growth by passing a protective trait onto the offspring.^85^ The mechanism of this intergenerational protection remains unidentified but is likely to be epigenetic in nature. Epigenetic mechanisms regulate gene activity in the absence of changes to the underlying DNA sequence, and include DNA methylation, histone modifications, chromatin-modifying proteins, and noncoding RNAs. These processes regulate how densely specific regions of DNA are compacted, thereby either preventing or enabling protein access, such as that of transcription factors, to DNA. More recently, it has been suggested that programmed effects can be observed beyond the first generation and that therefore epigenetic mechanisms could provide a vector of transmission of phenotype from parent to offspring across generations.^86^

Studies using the chicken embryo have also reported that incubation of fertilized eggs under hypoxic conditions recapitulates many components of the adverse cardiovascular phenotype described in rodent studies of chronic hypoxic pregnancy or in clinical studies of human pregnancy complicated by intrauterine growth restriction. Independent research groups have reported that chicken embryos incubated under hypoxic conditions show aortic wall thickening and nitric oxide–dependent endothelial dysfunction with enhanced sympathetic innervation in the peripheral vasculature, promoting a peripheral vasoconstrictor phenotype.^87–91^ Despite sensitized cardiac β-adrenergic receptors,^92^ hypoxic chicken embryos have reduced left ventricular ejection fraction and contractility, with diminished left ventricular developed pressure, indicative of uncompensated systolic dysfunction.^84,90–95^ Adult chickens raised from eggs incubated at high altitude show echocardiographic indices of pulmonary hypertension and right heart dysfunction.^96^ Adult birds incubated under hypoxic isobaric conditions but raised in normoxia after hatching develop systemic hypertension with evidence of increased cardiac sympathetic reactivity and cardiac wall thickening and enhanced contractile responses to sympathetic stimulation while impaired, circulating nitric oxide bioavailability and nitric oxide–dependent endothelial dysfunction in the peripheral vasculature.^88,93,97^ Itani et al^84,89,90^ have also reported that hypoxic incubation promotes oxidative stress, reduces the expression and activity of endogenous antioxidant enzymes, and impairs nitric oxide levels in the cardiovascular system of the chicken embryo at term. Further, treatment of chicken embryos with antioxidants (eg, melatonin) or agents that increase nitric oxide bioavailability (eg, pravastatin and sildenafil) rescues cardiovascular dysfunction during hypoxic development.^84,89,90,98^ Collectively, therefore, these studies in birds not only help to isolate the direct effects of chronic hypoxia on fetal cardiovascular development previously reported in mammals, including humans, but they also support that oxidative stress acting directly on the fetal cardiovascular system independent of the presence of a placenta is an important mechanistic driver of programming induced by developmental hypoxia, and that antioxidant therapy during the period of suboptimal development can be protective.

#### Insight From Ovine Studies

To further translate these concepts into the human clinical situation, work described in small rodents and birds should ideally be replicated in a larger mammalian experimental model that boasts cardiovascular developmental milestones comparable to those in humans, such as those in sheep.^99^ Pioneering studies of sheep undergoing pregnancy at high altitude, exposure to high ambient temperature, after carunclectomy, or during restriction of uteroplacental blood flow have reported striking similar effects on fetal growth and cardiovascular adaptations in fetal life.^100–105^ It has been harder to attribute many of these effects to those induced by isolated chronic fetal hypoxia during pregnancy or to investigate the longer-term consequences of hypoxic pregnancy for the cardiovascular health of adult offspring. Progress in this field of science using sheep has been held back by several technical barriers. First, there has been an inability to induce significant long-term reductions in fetal oxygenation to values similar to those measured in human pregnancy complicated by fetal growth restriction, without incurring concomitant reduction in other nutritional substrates for fetal growth and development. Second, there has been an inability to record continuous in vivo cardiovascular function from the mother and the fetus as the hypoxic pregnancy is actually occurring. Third, few studies have maintained cohorts of lambs born from hypoxic pregnancies and raised them under normoxic conditions to study the cardiovascular system of the offspring at adulthood. To address these gaps of knowledge, we have now created isobaric chambers able to maintain pregnant sheep for long periods of gestation under tightly controlled reductions in ambient oxygenation. This has been coupled with the design of a wireless data acquisition system which permits recording of several maternal and fetal blood pressure and blood flow signals via surgically-implanted catheters and flow probes in free-moving animals. Using this novel technology, we now know that hypoxic pregnancy in sheep of the type that mimics the reduced fetal oxygen levels in human complicated pregnancy recapitulates many of the effects on fetal growth, fetal cardiovascular function, and increased programmed cardiovascular risk in the offspring.^38,40,106–108^ Pregnant ewes, in contrast with pregnant rodents, are a much more resilient to adverse hypoxic environments. Therefore, this level of chronic hypoxia in sheep occurs without any significant reduction in maternal food intake and in the absence of sustained elevations in maternal or fetal stress hormones.^38,107^ The latter point is important as it highlights that hypoxic pregnancy can program cardiovascular dysfunction in the adult offspring independent of activation of the hypothalamic–pituitary–adrenal axis. Elegant studies of sheep undergoing pregnancy at high altitude by the group at Loma Linda are also consistent with the idea that hypoxic pregnancy can trigger a fetal origin of cardiovascular dysfunction, independent of excess fetal glucocorticoid exposure.^100^

Using the Cambridge model of chronic hypoxic pregnancy, Allison et al^40^ reported that simultaneous measurement of fetal Pao_2_ in the carotid and femoral arteries and carotid and femoral arterial blood flow within the hypoxic chambers permitted calculation of regional oxygen delivery in the chronically hypoxic sheep fetus. That study reported that the ratio of oxygen delivery to the fetal carotid circulation relative to the femoral arterial circulation increased significantly and progressively in the chronically hypoxic fetus. This confirms a maintained fetal brain-sparing hemodynamic response in the chronically hypoxic fetus. Shaw et al^106^ reported that fetal heart rate variability in sheep is reduced by chronic hypoxia because of dysregulation of the sympathetic control of the fetal heart rate. That study suggested a potential mechanism by which a reduction in indices of fetal heart rate variability predicts infants at increased risk of neonatal morbidity and mortality in humans. In this ovine model of hypoxic pregnancy, oxidative stress remains an important culprit mechanism and there is successful intervention with antioxidants.^107,108^ Brain et al^107^ reported that maternal treatment with vitamin C in ovine hypoxic pregnancy protected against oxidative stress by enhancing fetal antioxidant capacity and improving transplacental oxygenation.^107^ These effects of maternal treatment with vitamin C in hypoxic pregnancy protected against fetal growth restriction and normalized nitric oxide bioavailability, peripheral vascular function, and arterial blood pressure in adult offspring. Botting et al^108^ more recently reported that the beneficial effects of maternal vitamin C treatment during hypoxic pregnancy in sheep on fetal growth and programmed hypertension in adult offspring could be replicated and improved by maternal treatment with MitoQ in hypoxic pregnancy. These findings are important because MitoQ is a mitochondria-targeted antioxidant with enhanced human translational capacity. Specifically, compared with conventional antioxidants, MitoQ protects against cardiovascular dysfunction in adult offspring programmed by hypoxic pregnancy, without opposing the fetal vascular oxidant tone that contributes to the fetal redistribution of blood flow during hypoxic conditions (Figure 3).^108^ This is extremely important, as it means that use of MitoQ in hypoxic pregnancy can protect the fetus against programmed systemic hypertension at adulthood, without weakening the fetal brain-sparing response to hypoxia.^108^ In recent years, several magnetic resonance imaging approaches for the assessment of placental function and fetal cardiovascular physiology in sheep have also been developed.^109^ These noninvasive techniques provide indispensable approaches to identifying placental and fetal cardiometabolic dysfunction in complicated pregnancy. Such technologies hold significant promise which will help in the translation of potential interventions, such as the use of mitochondria-targeted antioxidants, into the human clinical situation.

### Conclusions

In this review, data derived from clinical studies, as well as from a variety of animal models, have been compiled to support a strong link between chronic fetal hypoxia and increased susceptibility to cardiovascular dysfunction in the offspring (Table). Babies born from pregnancies complicated by chronic fetal hypoxia have an elevated risk of fetal growth restriction, endothelial dysfunction, sympathetic hyperinnervation, and alterations in the structure and function of the heart and vessels that increases susceptibility to cardiovascular dysfunction in later life. Descriptive studies in this field have now been extended with highly mechanistic investigations, aiming to isolate underlying signaling pathways to identify potential therapy. Studies from many independent laboratories now proposes oxidative stress in the placenta and developing fetal organs as a major mechanism linking suboptimal pregnancy with fetal growth restriction and an increased cardiovascular risk in the adult offspring (Table). Oxidative stress decreases nitric oxide bioavailability, impairing perfusion and thereby the delivery of oxygen and nutrients to the developing conceptus. Maternal treatment with antioxidants in pregnancy complicated by oxidative stress restores nitric oxide bioavailability protecting perfusion. Therefore, in contrast with other treatments, such as maternal hyperoxygenation in pregnancy complicated by fetal growth restriction, maternal antioxidant therapy restores the delivery of all essential nutrients necessary for appropriate fetal growth and development. Initial studies of maternal treatment with the antioxidant vitamin C in hypoxic pregnancy have provided the necessary proof-of-principle findings to support that maternal antioxidant therapy can protect against fetal growth restriction and programmed cardiovascular dysfunction in adult offspring. More recent studies have enhanced the human translational potential of these ideas, showing that maternal treatment with the mitochondria-targeted antioxidant MitoQ in hypoxic pregnancy can offer improved protection on fetal growth and programmed heart disease in the adult offspring. In contrast with conventional antioxidants, maternal treatment with MitoQ protects against developmental programming of heart disease in adult offspring while maintaining the fetal brain-sparing response to chronic hypoxia, thereby ensuring concomitant protection of the developing cardiovascular and central nervous systems in progeny. Future studies should focus on the applicability of mitochondria-targeted therapy to the human clinical situation, evaluating its safety for mother and child.

**Table.**
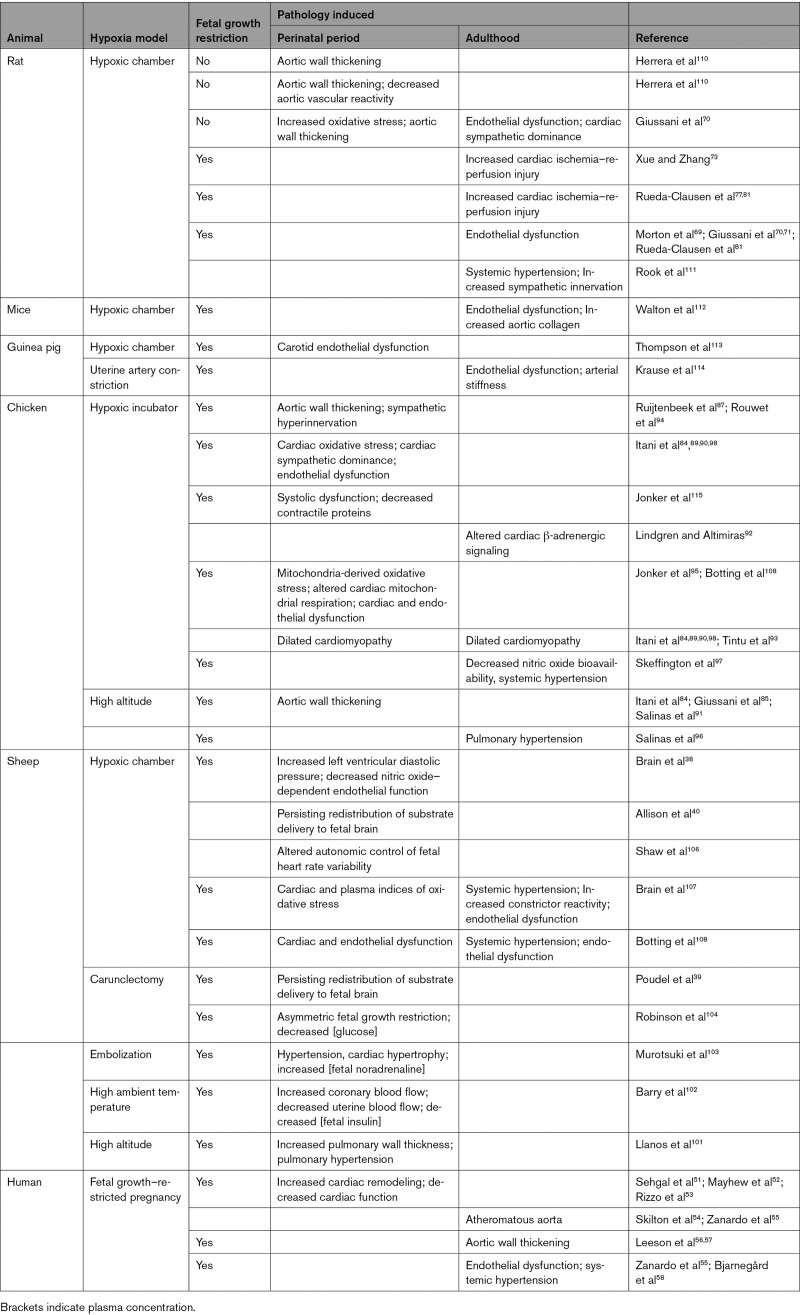
Consequences of Prenatal Hypoxia in Humans and Experimental Animals

## Acknowledgments

Dino Giussani is Professor of Developmental Cardiovascular Physiology and Medicine in the Department of Physiology, Development, and Neuroscience at the University of Cambridge; Professorial Fellow and Director of Studies in Medicine at Gonville and Caius College; a Lister Institute Fellow; and a Royal Society Wolfson Research Merit Award Holder.

## Sources of Funding

This work is supported by The British Heart Foundation (RG/17/8/32924) and the Medical Research Council UK (MR/V03362X/1). Both programs of research support Dr Giussani.

## Disclosures

None.
